# Posture and Time Arrangement Influence Shank Circumference Reduction When Performing Leg Raising Exercise

**DOI:** 10.3390/ijerph17165735

**Published:** 2020-08-08

**Authors:** Yi-Lang Chen, Ya-Ting Cheng, Jia-Ning Ye, Tzu-Ling Huang, Wen-Ning Chen

**Affiliations:** 1 Department of Industrial Engineering and Management, Ming Chi University of Technology, New Taipei 24301, Taiwan; m08218010@o365.mcut.edu.tw (Y.-T.C.); m08218008@o365.mcut.edu.tw (J.-N.Y.); m08218012@o365.mcut.edu.tw (T.-L.H.); m08218013@o365.mcut.edu.tw (W.-N.C.); 2 Department of Industrial Design, Chang Gung University, Touyuan 33302, Taiwan

**Keywords:** leg raising, holding posture, shank circumference (SC), subjective discomfort

## Abstract

This study recruited nine young women who performed a leg raising exercise under six test combinations of three holding postures (lying supine, placing the shanks on a yoga ball, and elevating the legs at 60° against the wall) and two time arrangements (continuous vs. intermittent) for a period of 15 min. The intermittent arrangement included an additional 1-min rest set in the middle of the 15 min test. The participants’ shank circumference (SC) reductions and discomfort ratings were measured after each test combination was performed. Results indicated that the most efficient method for SC reduction was the yoga ball (5.4 mm), followed by the supine lying posture (4.6 mm) and wall-supported leg raising (3.1 mm). A significant interaction of posture × time arrangement (*p* < 0.01) implied that the yoga ball method combined with a 1-min rest produced the greatest SC reduction (6.7 mm). Our results also showed that participants experienced the greatest discomfort (score: 4.96) when performing wall-supported leg raising, compared with both lying supine (score: 1.34) and the yoga ball (score: 1.32). This study suggests that the effectiveness of leg raising as conventionally practiced for eliminating leg fatigue or swelling requires further consideration.

## 1. Introduction

Previous investigations have indicated that prolonged standing and daily activity are likely to contribute to musculoskeletal and vascular symptoms. The symptoms include muscle fatigue, lactic acid accumulation, swelling, and soreness in human lower limbs [1,2,3,4]. Many solutions have been proposed to effectively reduce these leg symptoms, such as using floor mats [2] or graduated compression/elastic stockings [5,6], appropriate work–rest cycles [7] and static stretching [8], and intermittent walking [9]. However, these maneuvers are limited because most of them are related to the organizational strategies/regulations and are intervened directly in people’s works and activities.

The limitations of these methods make it feasible to exercise afterwards. One of the most popular methods is leg raising exercise because the exercise can be carried out by individuals. To eliminate leg fatigue and swelling, in Taiwan, many young women usually perform leg raising exercise while lying on a bed; the bedside wall supports the heels, and there is a widespread perception that leg raising before bedtime can prevent varicose veins on the legs [10]. In addition, this exercise is also employed by many people (e.g., young women) for aesthetic purposes.

In clinical medicine, the aforementioned leg raising is called passive leg raising (PLR). As early as 1881, Forst described a leg raising test to provoke pain in patients with sciatica [11]. The test is one of the methods currently used to predict the response of cardiac output to pretreatment fluid administration in patients with acute respiratory distress syndrome [12,13]. It involves moving the patient from a semi-recumbent position to one in which the trunk is horizontal and the inferior limbs are passively elevated at approximately 45° [14,15,16]. Passive leg raising also shifts blood from the lower extremities toward the intrathoracic compartment [17]. Because leg raising is a simple and effective maneuver, it has recently attracted increasing interest for application in various tests for monitoring heart function [18], assessing fluid responsiveness [19], and unmasking pulmonary hypertension [20]. However, before its popularity in fluid responsiveness tests, it was a rescue maneuver that had been used for years by first-aid rescuers. Zhang et al. [18] suggested that PLR could improve cerebral and coronary perfusion and be a beneficial supplement to cardiopulmonary resuscitation (CPR), as it is not necessary to lift the legs too high above the ground. When the legs were lifted to 30°, 45°, 60°, and 90° from the ground, the volumes transferred from the legs to the upper body were 36%, 43%, 47%, and 50% of the initial volume in the legs, respectively.

Even though the PLR test has been well examined and its benefits have also been verified clinically, the effect of leg raising on fatigue and swelling of the lower limbs has received less attention, especially for healthy adults. Because of limited knowledge regarding leg raising from an ergonomic perspective, inappropriate raising or incorrect posture may cause physical discomfort or even pain. Unfortunately, no previous research addressed this issue. To fill the gap, we recruited nine young women as participants, who were instructed to perform leg raising under six test combinations of three postures and two time arrangements. The participants’ shank circumference (SC) reductions, as well as the discomfort ratings after performing each test combination were examined. We attempted to compare the effectiveness of these leg raising postures with different time arrangements on SC reduction.

## 2. Materials and Methods

### 2.1. Participants

This study initially recruited twelve female university students as participants. Recruitment information was announced on the bulletin board of Ming Chi University of Technology (New Taipei, Taiwan) during 1–15 December, 2019. Included were female university students older than 18 years who partook in regular and normal daily activities. An interview was conducted for each participant with special attention to the daily activities prior to the experiment. All participants were familiar with the experimental procedure of this study and were also informed of the nature of the study and did not allow their sleeping, eating, and drinking habits to change throughout study participation. To eliminate possible pathology in the participants, a short medical history was taken with special attention to complaints concerning the lower back and musculoskeletal injuries. Out of the total of 12 students, three could not participate or complete the experiment; two students were excluded because of overweight [2] and one student was because of calf cramps during the experiment. Consequently, nine healthy female students completed the experiment. Among them, five had a habit of leg raising and four did not. Their mean (standard deviation (SD)) age, height, and body weight were 23.1 (1.7) years, 161.0 (4.0) cm, and 60.0 (11.5) kg, respectively. The experimental procedures were approved by the Ethics Committee of Chang Gung Memorial Hospital, Taiwan (ethical code: No. 983653). All study participants provided written consent prior to the experiment and received remuneration.

### 2.2. Experimental Design

**Figure 1:**
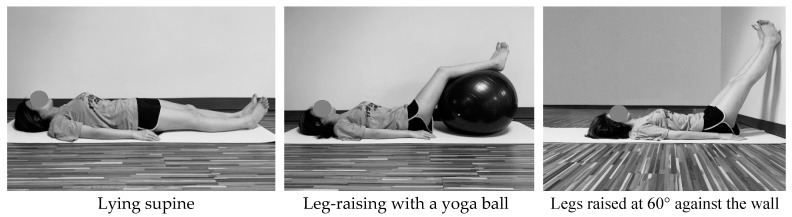
Three types of leg raising postures performed by each participant.

We examined the effect of leg raising posture and time arrangement on SC reduction for varying test combinations. Three types of holding posture included lying supine, legs raised by a yoga ball (65 cm in diameter), and lifting the legs 60° against the wall (Figure 1); a 60° incline was determined to be the angle adopted by most young Taiwanese women who practice leg raising. The second independent variable comprised two types of time arrangement. In the continuous method, legs were kept raised for 15 min without rest, whereas in the intermittent method, the raising period was also set at 15 min, but with an additional 1-min rest in a supine posture after 7.5 min. However, the participants were requested to maintain the original posture for the 1-min rest in the intermittent condition when performing the supine lying posture. To obtain a basis for comparison, SC values at three time points (after getting up and before and after performing the leg raising test) were collected for each participant. All participants rated subjective discomfort in the lower limbs once each leg raising test was completed. For the analysis, the post-test measurement was subtracted from the pre-test measure to calculate the reduction in SC following each of the six test interventions. Hence, a total of 54 SC reduction and 54 discomfort scores were collected (9 participants × 3 postures × 2 time arrangements) in a random order throughout the experiment.

### 2.3. Experimental Procedure

Prior to the experiment, the participants’ anthropometric measurements including age, stature, and body weight were recorded. The six test combinations of each participant were carried out in the random order and were arranged within a period of 2 weeks, with an average of approximately one trial every two days for each participant. The experiment was performed regularly between 20:00 and 21:00 every night in a female dormitory. In addition, the SC of each participant was also measured every day after getting up and before the experiment for comparisons. After each test combination was completed, the participants’ SCs and subjective discomfort of the lower limbs were recorded. On the basis of the SC measurements proposed by previous studies [2,3], we used a pull–push tester (MP-1, Attonic, Japan) to control the fixed force applied during the measurement to reduce errors. We measured the participants’ SCs at the midpoint between the knee and ankle joints (the point was marked during the whole testing period). When measuring the SC, participants were requested to stand on the ground to minimize variation. Each SC measurement was repeated three times and the average of the two closest values was then used for further analyses.

In this study, the subjective assessments of lower limb discomfort were performed using a continuous visual analogue scale (VAS) [21]. The scale was 100 mm in length and was modeled after the comfort scales developed by Mündermann et al. [22]. The VAS has been reported to be a reliable assessment of perception and is more precise than an ordinal scale that ranks responses [22]. The left end of the scale was labeled no discomfort at all and the right end was labeled extreme discomfort. The participants used a pen to complete ratings by marking locations along the scale that most accurately represented their feeling of discomfort after a trial. An analyst used a ruler to measure the distance from the no discomfort at all anchor to the location of a mark, and data were measured for analysis.

### 2.4. Statistical Analyses

The difference in the participants’ measured SC was determined by calculating the SC reduction after performing the different test interventions (3 postures—lying supine, placing their feet and shanks on a yoga ball, and elevating the legs 60 ° against the wall) × 2 time arrangements (continuous vs. intermittent) relative to the SC measured before the experiment. A two-way repeated measures analysis of variance (RM ANOVA) was used to process the experimental data (SC reduction and discomfort score). Each participant was considered as a block and performed all treatment combinations in a random order. Significant independent variables were further analyzed with Duncan’s multiple range test (Duncan MRT). Moreover, a paired *t* test was used to assess whether the SC values among the three measurement sessions were significantly different. A power value was used to examine if the effect size of any significant independent variable was satisfactory (i.e., power ≥ 0.8) or not [23]. All statistical analyses were performed with SPSS 22.0 statistical software (IBM Corp.: Armork, NY, USA), with a significance level of *α* = 0.05 for all tests.

## 3. Results

### 3.1. Posture Effect on SC Reduction

**Figure 2:**
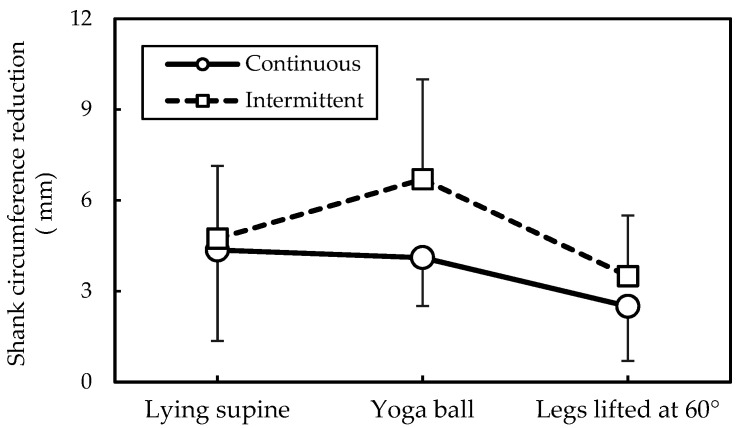
Interaction of posture and time arrangement on shank circumference reduction.

The paired *t* test showed that after around 12 h of daily activities, the participants’ SCs increased significantly with an average of 7.8 mm (*t* = −10.93, *p* < 0.001). As shown in Table 1, all measured SC values differed significantly between any two measurement sessions (all *p* < 0.001). The two-way ANOVA (Table 2) indicated that different postures affected SC reduction (*p* < 0.05), whereas the Duncan MRT revealed significant differences in SC reduction between the yoga ball condition and wall-supported leg raising (Table 3). That is, legs raised on yoga ball caused the most effective SC reduction (5.4 mm), whereas Legs raised 60° against the wall exhibited a worst result (3.1 mm). Although time arrangement had no significant effect on SC reduction, the interaction of posture and time arrangement was significant (*p* < 0.01, Table 2), indicating that a cross analysis was warranted. As shown in Figure 2, the effect of time arrangement on SC reduction was more significant when legs raised on a yoga ball (+2.6 mm) than against the wall (+1.0 mm).

### 3.2. Subjective Discomfort Results

The ANOVA results for subjective discomfort rating (Table 4) indicated that posture significantly influenced the discomfort score (*p* < 0.001). The Duncan MRT (Table 5) further revealed that participants experienced the greatest discomfort with wall-supported leg raising (score: 4.96) compared to the yoga ball (score: 1.32) and supine lying posture (score: 1.34). No difference was observed between the conditions of the yoga ball and lying supine, as shown in Table 5.

## 4. Discussion

Although leg raising test has been developed for clinical applications, the test is also currently used for eliminating shank stresses in healthy adults before bedtime. However, the effect of leg raising on fatigue and swelling elimination of the lower limbs is still unclear. This study therefore recruited nine young women to perform leg raising under six test combinations of three postures (namely lying supine, placing their feet and shanks on a yoga ball, and elevating the legs 60° against the wall) and two time arrangements (continuous vs. intermittent). The participants’ SC reductions and the discomfort ratings after performing each test combination were examined. Our results showed that leg raising on a yoga ball, including a 1-min rest in the middle of a 15-min period, produced the greatest reduction of SC among the six test combinations. However, simply lying supine produced a similar effect. Surprisingly, the leg raising exercise preferred by many young women (raising the legs against the wall at an angle) was not as effective as expected.

Results revealed that the SC values of three time sessions differed significantly from each other (Table 1). The participants’ SCs increased significantly, with an average of 7.8 mm from morning to night. This may represent an accumulated leg strain (e.g., shank region) for normal daily activity. The increase in SC observed in this study was somewhat higher than the data (7.1 mm) obtained by Chen et al. [3], where the participants were requested to remain standing still for 2 h. Table 1 also shows that the participants’ SCs decreased significantly after the leg raising test. However, their SCs still did not recover to the basic level seen in the morning; on average, only approximately 60% SC reduction was achieved. This result implies that leg raising exercise sustained over a 15-min period may be insufficient; how long is necessary for recovery to the original basis requires further investigation. However, it also depends on different leg raising maneuvers.

Leg raising posture how to influence the SC reduction was of interest in this study. As shown in Table 2, the two-way ANOVA indicated that posture variable significantly affected SC reduction (*p* < 0.05). The most efficient method for reducing SC was yoga ball usage (5.4 mm), followed by supine lying posture (4.6 mm), and wall-supported leg raising at 60° (3.1 mm). However, time arrangement had no significant effect on SC reduction. Because interaction effect of posture and time arrangement on SC reduction was significant, a cross analysis result showed that effect of time arrangement on SC reduction was dependent on what posture was employed; the effect was more notable when legs raised on a yoga ball (+2.6 mm) than against the wall (+1.0 mm), with a difference more than two times (Figure 2). Our results further showed that raising the legs on a yoga ball with an intervening 1-min rest produced the greatest SC reduction (6.7 mm), whereas continuous wall-supported leg raising produced the least SC reduction (2.5 mm), indicating SC recovery rates of about 86% and 32%, respectively (Figure 2). In addition, because the participants were requested to maintain the original posture for the 1-min rest in the intermittent condition, there was almost no time arrangement effect on SC reduction when performing the supine lying posture, as shown in Figure 2.

Our present analyses imply that a short time break caused significantly different results. In comparison with this study, Chen et al. [3] examined the effect of a prolonged 2-h standing period on SC increase and found that when participants stood for 20 min and relaxed for 1 min, the SC increase was reduced overall by approximately 40%. In other words, with only a short resting time, the participants could more effectively alleviate the load exerted on their legs. In this study, a similar phenomenon was observed with the legs raised on a yoga ball with a 1-min rest. This may be explained by a massage-like effect. Improved blood flow is a perceived benefit of massage and includes sustained reductions in muscle tightness [24,25]. An intervening 1-min rest in leg raising with a yoga ball may accelerate blood flow in the legs due to the compress–relax–compress pattern, while the identical posture without rest statically compresses both the shanks and yoga ball, thus relatively slowing blood flow. Our present results showed no significant differences in SC reduction between the supine lying posture and yoga ball leg raising or between lying supine and wall-supported leg raising. Hence, a supine lying posture may also be effective for reducing SC; by contrast, wall-supported leg raising, which caused greater discomfort (score: 4.96) compared with the others (score: ~1.33), may be impractical. During wall-mounted leg raising, the participants’ heels were supported by the bedside wall, whereas their unsupported legs were hanging. In clinical practice, by contrast, the legs are raised and supported using the bed adjustment to avoid touching the patient and to minimize pain [26]. These two postures are effectively quite different. Given the current study design and dependent variables measured, it is difficult to discern why wall-supported leg raising was ineffective, and further clarification is required.

Although the similar leg raising is performed, the purposes are quite different between clinical treatment and fatigue relief; the user populations are also different (patients vs. healthy adults). Leg raising exercise used by healthy population before bedtime is based mainly on a common intuition that raising the legs can help reduce leg fatigue and swelling, thus maintaining a slim shank shape. In addition to this aesthetic purpose, the exercise is also popular among employees who require prolonged periods of standing (e.g., nurses, salesclerks, and guards). Although few studies have considered that such exercise may improve symptoms in some patients with varicose veins [10], the effectiveness of different raising postures for eliminating leg fatigue or swelling should be carefully identified.

This study has several limitations. One major limitation of this study is the relatively small and single-sex sample (9 young women with a mean age of 23.1 years) recruited in the test. Table 2 and Table 4 also shows that the 9 participants exhibited statistical powers ranging from 0.706 to 0.991, with a 0.05 alpha level for the test combinations used in this study. The power value of ≥0.8 is generally accepted in the analysis as suggested by Cohen [23]. This indicates that the effect of posture on SC reduction may require further clarification through a larger sample size because the power value was somewhat less than 0.8 (i.e., 0.706), even though the effect of interaction of posture and time arrangement exhibited a high power value (i.e., 0.926). In this study, all the participants were young women, so the results may not be applicable to men. Another concern is that only a single objective index (i.e., SC reduction) was employed for assessing the effects of leg raising. Other clinical responses, such as volume transferred from the legs to the upper body, cardiac output, coronary perfusion pressure, and blood flow to the heart [18], may alter interpretation of the results. Moreover, this study set the leg raising time for 15 min and the position at 60° based on our pilot survey; a longer testing time may yield different results. These limitations should be considered before applying the results.

## 5. Conclusions

This study examined the effects of leg raising posture and time arrangement on participants’ SC reduction, because the leg raising exercise is widely employed among young Taiwanese women. Results revealed that raising posture affected the SC reduction and discomfort score. This study suggested that for a 15-min leg raising exercise, using a yoga ball with a short intervening rest is most effective for SC reduction. In practice, simply lying supine could produce a similar effect. Surprisingly, the leg raising exercise preferred by many young women (raising the legs against the wall at an angle) was not as effective as expected. The findings suggest that the effectiveness of leg raising exercise as conventionally practiced for eliminating leg fatigue and swelling should be further examined.

## Figures and Tables

**Table 1:** Results of paired *t* test for different SC measurement sessions.

Session	SC (mm)	After Getting Up	Before Leg Raising
After getting up	342.6 (32.6)	---	---
Before leg raising	350.4 (32.0)	*t* = −10.93, *p* < 0.001	---
After leg raising	345.7 (31.5)	*t* = −4.45, *p* < 0.001	*t* = 10.46, *p* < 0.001

*Notes:* SC—shank circumference; Data in mean (standard deviation).

**Table 2:** Two-way ANOVA results of SC reductions.

Source	DF	SS	MS	F	Significance	Power
Posture	2	0.544	0.272	4.164	*p* < 0.05	0.706
Time arrangement	1	0.112	0.112	1.711	*p* = 0.197	0.249
Posture × Time arrangement	2	0.968	0.484	7.417	*p* < 0.01	0.926

*Notes:* SC—shank circumference; DF—degree of freedom; SS—sum of square; MS—mean square; F—F value; power, effect size.

**Table 3:** Duncan MRT results of SC reduction under various leg-raising postures.

Holding Posture	Observations	SC Reduction (mm)	Duncan Groups
Lying supine	18	4.56 (2.75)	AB
Legs raised on yoga ball	18	5.36 (3.72)	A
Legs raised 60° against the wall	18	3.11 (1.87)	B

*Notes:* SC—Shank circumference; Data in mean (standard deviation).

**Table 4:** Two-way ANOVA results of discomfort scores.

Source	DF	SS	MS	*F*	Significance	Power
Posture	2	139.328	69.864	11.62	*p* < 0.001	0.991
Time arrangement	1	1.185	1.185	0.20	*p* = 0.659	0.072
Posture × Time arrangement	2	3.774	1.887	0.31	*p* = 0.732	0.097

*Notes:* DF—degree of freedom; SS—sum of square; MS—mean square; F—F value; power, effect size.

**Table 5:** Duncan MRT results of discomfort scores under various leg-raising postures.

Holding Posture	N	Discomfort Scores	Duncan Groups
Supine lying	18	1.34 (1.76)	A
Legs raised on yoga ball	18	1.32 (1.52)	A
Legs lifted 60° against the wall	18	4.96 (3.08)	B

*Notes:* Data in mean (standard deviation).
